# What does a “good life” mean for people living with dementia? A protocol for a think-aloud study informing the value of care

**DOI:** 10.3389/fnagi.2022.1061247

**Published:** 2022-12-16

**Authors:** Irina Kinchin, Iracema Leroi, Sean P. Kennelly, Slavica Kochovska, Conor Brady, Deborah Fitzhenry, Cathy McHale, Philip Kinghorn, Joanna Coast

**Affiliations:** ^1^Centre for Health Policy and Management, Trinity College Dublin, The University of Dublin, Dublin, Ireland; ^2^Improving Palliative, Aged and Chronic Care through Clinical Research and Translation (IMPACCT) Centre, University of Technology, Sydney, NSW, Australia; ^3^Global Brain Health Institute, Trinity College Dublin, The University of Dublin, Dublin, Ireland; ^4^Institute of Memory and Cognition, Tallaght University Hospital, Dublin, Ireland; ^5^Department of Medical Gerontology, Trinity College Dublin, The University of Dublin, Dublin, Ireland; ^6^Faculty of Science, Medicine and Health, University of Wollongong, Wollongong, NSW, Australia; ^7^Age Related Health Care Outpatient Services, Tallaght University Hospital, Dublin, Ireland; ^8^Memory Assessment and Support Service, Tallaght University Hospital, Dublin, Ireland; ^9^Health Economics Unit, Institute of Applied Health Research, University of Birmingham, Birmingham, United Kingdom; ^10^Bristol Population Health Science Institute, University of Bristol, Bristol, United Kingdom

**Keywords:** dementia, outcome measurement, preference-based health-related quality of life, wellbeing, capability approach, interview, economic evaluation

## Abstract

**Introduction:**

Economic evaluation currently focuses almost exclusively on the maximization of health, using the Quality-Adjusted Life-Year (QALY) framework with instruments such as the EQ-5D, with a limited number of health-focused dimensions providing the assessment of health benefit. This evaluative framework is likely to be insufficient for setting priorities in dementia care because of its exclusive concern with health. Data are also often collected from the perspective of a proxy, limiting the voice of those living with dementia in decision-making. This protocol describes a research project that aims to gather the perspectives of people living with dementia, their insights, and preferences for assessing their quality of life to inform economic evaluation outcome measurement and design with a goal of creating a more robust evidence base for the value of healthcare services. Specifically, this study will elucidate what a “good life” means to people living with dementia and how well instruments currently used in economic evaluation meet this description. This project will further test the acceptability of capability wellbeing instruments as self-report instruments and compare them to generic and dementia-specific preference-based instruments.

**Methods and analysis:**

People living with dementia, diagnosed, or waiting to receive a formal diagnosis and with the capacity to participate in research, will be invited to participate in an hour “think aloud” interview. Participants will be purposefully selected to cover a range of dementia diagnoses, age, and sex, recruited through the integrated care, geriatric, and post-diagnostic clinics at St James’ and Tallaght University Hospitals and dementia support groups in the Ireland. During the interview, participants will be invited to reflect on a “good life” and “think aloud” while completing four economic quality of life instruments with a perspective that goes beyond health (AD-5D/QOL-AD, AQOL-4D, ICECAP-O, ICECAP-SCM). An interviewer will then probe areas of difficulty when completing the instruments in a semi-structured way. The analysis will identify the frequency of errors in comprehension, retrieval, judgment, and response from verbatim transcripts. Qualitative data will be analyzed using constant comparison.

**Ethics:**

The St James’s Hospital and Tallaght University Hospital Joint Research Ethics Committee approved the study (Approval Date: 11 April 2022).

## 1 Introduction

The challenging questions of how healthcare systems can address the needs of patients efficiently and deliver equitable and cost-effective care remain ever pertinent as we continue navigating through the COVID-19 pandemic. The field of health economics and outcomes research offers tools to conduct clinical and economic evaluations of health interventions. These tools serve as an essential foundation to derive information on efficiency and value across the healthcare system. While these evaluations are vitally important, the experience of the past years has surfaced the importance of patient-reported outcome measures (PROMs) to inform investment in services and support person-centered and value-based care by providing a way of measuring health outcomes from the patient’s perspective (Churruca et al., 2021).

Patient-reported outcome measures provide an opportunity for people living with dementia to share through questionnaires their perceptions of health and wellbeing, quality of life, daily functioning, and symptoms, as well as experiences of care. Responses to PROMs questions enable healthcare services to tailor the care that patients need and want. These measures aim to fill a vital gap in the knowledge about outcomes that matter to people living with dementia.

A number of dementia-specific PROMs exist, yet none are used in dementia registries, and the majority of studies utilize PROMs *via* a proxy (Ayton et al., 2020). Measuring the quality of life of people with dementia is complex. Despite strong indication within the literature that people with dementia are willing (Hirschman et al., 2005) and able to express views and participate in decision-making (Kim et al., 2002) as well as respond consistently to questions about preferences and choices related to daily living (Feinberg and Whitlatch, 2001; Kane et al., 2003; Moyle et al., 2012a), their participation is often limited (Tyrrell et al., 2006; Fetherstonhaugh et al., 2013; Daly et al., 2018).

Denied or tokenistic participation in decision-making could be attributed to several reasons. Traditionally, PROM data is collected using conventional techniques, including surveys, questionnaires, or interviews, all of which require adequate cognitive and communication skills. Dementia may lead to progressive difficulties with memory, expressive and receptive language, and reading comprehension (Caramelli et al., 1998), making the traditional PROMs increasingly difficult for people living with dementia to complete. Consequently, proxies, family members or care partners are asked to complete the assessment on their behalf. While proxy ratings are considered valid, they are based on observable behavior rather than subjective experience. This mismatch of views could cause significant disparities in rating between the proxy and the person’s experiencing the condition (Karlawish et al., 2008; Gräske et al., 2012; Moyle et al., 2012b; Arons et al., 2013; O’Shea et al., 2020).

Augmentative and alternative communication (AAC) is a set of tools and strategies used to support people with multiple and complex cognitive and physical difficulties. The AAC methods provide alternative access to the preferences of people living with dementia in the form of single pages and phrases, pictorial tools, and other visual aids. The most recent literature review by Haroon et al. (2022) confirmed that quality of life information could be elicited more effectively from people living with dementia through pictorial tools. Yet, the application of the AAC methods to economic evaluation outcomes measurement and design is limited (Broomfield et al., 2019).

Another set of reasons for the complex nature of the PROM assessment in dementia could be attributed to the choice of instruments used to assess this construct. The EQ-5D is the most frequently chosen instrument in economic evaluations internationally (Szende et al., 2014). In the EQ-5D, the quality of life is defined by mobility, self-care, usual activities, pain/discomfort, and anxiety/depression. Hence, the EQ-5D assesses only physical and psychological aspects of quality of life and does not capture many critical psychosocial aspects that may influence the quality of life of people with dementia (Reilly et al., 2020).

Recent studies on how people with dementia might live well with the condition indicate that factors beyond health for example, self-efficacy and humor contribute significantly to their overall quality of life (Clarke et al., 2020; Lamont et al., 2020). Economic quality of life assessment could be incomplete without accounting for elements like the quality of personal and social relationships, effective communication, feeling valued and respected by others, and independence (Reilly et al., 2020). Instruments such as the EQ-5D may not capture aspects of quality of life beyond health that people living with dementia themselves consider essential.

An alternative approach has been proposed to capture broader wellbeing called the capability approach. Sen (1987) developed the capability approach with a core focus on what individuals are free and able to do and be (i.e., are capable of). It is a theoretical framework with a growing range of applications (Helter et al., 2020). For example, it has been highlighted as a way of helping people with mental health difficulties engage with their values and priorities, for instance, by influencing the design and delivery of mental health services (Helter et al., 2022).

Capability instruments like the ICECAP for Older people (ICECAP-O) and ICECAP Supportive Care Measure (ICECAP-SCM) have been developed to supplement the EQ-5D and are increasingly used in economic evaluations and trials, particularly among older people. The ICECAP-O uses a broader scope of quality of life domains identified by older people as important to wellbeing (attachment, security, role, enjoyment, and control) (Coast et al., 2008). The ICECAP-SCM covers seven domains (love and affection, choice, physical suffering, emotional suffering, dignity, support, and preparation) (Bailey et al., 2016).

Assessment of the quality of life of people living with dementia using the capability wellbeing approach is emerging (Helter et al., 2020). To date, two studies have elicited responses directly from community-dwelling people living with dementia using the ICECAP-O (Nyman et al., 2021; Bibi et al., 2022). In comparison, three other studies surveyed proxies using either ICECAP-O (Makai et al., 2014; Sarabia-Cobo et al., 2017) or ICECAP-O and ICECAP-SCM (Froggatt et al., 2020). The ICECAP-O was found to be both a reliable and valid measure of the quality of life for use with people living with mild/moderate dementia without a proxy (Nyman et al., 2021; Bibi et al., 2022). It is not entirely clear yet, however, which ICECAP (ICECAP-O for older people or ICECAP-SCM for those at the end of life) is to be used when assessing the quality of life of people living with dementia; or whether a dementia-specific capability wellbeing instrument(s) co-designed with people living with dementia is warranted to support the evidence base for the effectiveness and value of healthcare services at different stages and within the distinct contexts in which they experience life.

### 1.1 Aims

The overarching aim of this study is to gather perspectives of people living with dementia, their insights, and preferences on the question of assessing their quality of life in economic evaluation. Specifically, this study aims to address two research questions (RQ):

**RQ1:** What does a “good life” mean to someone living with dementia?

**RQ2:** How well do instruments currently used in economic evaluation reflect this construct?

Four different types of preference-based PROMs with a perspective that goes beyond health will be tested in this study. These comprise a dementia-specific quality of life PROM (*AD-5D/QOL-AD*), a generic health-related quality of life PROM (*AQOL-4D*), and two capability PROMs of broader wellbeing (*ICECAP-O* and *ICECAP-SCM*).

Specific objectives of this study include:

(1) To define themes of a “good life” or quality of life from the perspective of people living with dementia as well as explore differences and similarities in the type of dementia, age and sex.
(2) To gather perspectives of people living with dementia, their insights, and preferences for assessing their quality of life using different preference-based patient-reported instruments, their design and administration mode.
(3) To explore differences in the completion of the PROMs in terms of *comprehension* (e.g., any misunderstanding of a word, phrase, or response option); *retrieval* (e.g., a recall error); *judgment* (e.g., recalled experiences are irrelevant or inadequate); *response* (e.g., participant’s response is inconsistent with the personal experience expressed or the desired response is missing from the response choices); and *completion time*.

## 2 Methods and analysis

This protocol adheres to the Consolidated Criteria for Reporting Qualitative Studies (COREQ) (Tong et al., 2007).

### 2.1 Theoretical framework

This research project comprises a “think-aloud” study followed by a semi-structured interview. A think-aloud study is a cognitive interview method that asks participants to verbalize their thoughts and actions as they perform a task (Willis, 2005). Think-aloud interviews enable examining comprehension, retrieval, judgment, and response difficulties when completing a questionnaire. The interviewer remains silent so long as individuals continue to think aloud. This process allows for a more realistic picture of the problems that individuals face when completing questionnaires than more direct interview methods that may interrupt task completion (Kuusela and Paul, 2000).

### 2.2 Participant selection and setting

#### 2.2.1 Sampling

A purposeful sample will be recruited for this study. Potential participants will be recruited through the integrated care, geriatric and post-diagnostic clinics at Tallaght University Hospital and St James’ Hospital in Dublin, Ireland, and supplemented with additional recruitment *via* dementia support groups. Participants will be required to satisfy the following inclusion criteria:

• being diagnosed or waiting to receive a formal diagnosis of dementia;
• have the capacity to participate in research, i.e., sufficiently well to be able to provide informed consent and participate in research;
• wishing to participate; and,
• able to communicate in the English language.

Patients who are medically or psychiatrically unstable or acute, as assessed by the clinical team, will not be eligible to participate. All of the inclusion criteria must be satisfied for a person to be eligible to participate.

#### 2.2.2 Method of approach

For clinical recruitment, a clinician, during a routine appointment, will approach an eligible patient with an opportunity to participate in this study. If interested, potential participants will be handed a participant information leaflet to read in their own time. If they agree to participate, they can leave their contact details (name, email address, or phone number) with a research team member present at the time or contact the research team directly to discuss the study further, obtain informed consent and arrange for study participation.

For recruitment *via* dementia support groups, a group facilitator would approach potential participants using their judgment of the person’s capacity to participate in research and ability to provide informed consent. They will approach potential participants with an invitation to participate in the study. If interested, potential participants will be given a participant information leaflet to read in their own time. If they are interested in participating, they can leave their contact details with the group facilitator or contact the research team directly.

#### 2.2.3 Setting of data collection and consent

Interviews will be completed at a time and place convenient for the participants, either face-to-face in the participant’s home or any other convenient location or remotely *via* platforms such as Teams. If the participant opts for a home visit, a research team member will revisit the information in the participant information leaflet, and proceed to obtain written, informed consent from the participant. If the participant opts for remote participation, the consent forms will be sent by email or post at least 1 week before the interview. Once connected remotely, a research team member will go through the consent form and the participant information sheet and address any questions relevant to participation. If agreeing to consent, participants will be asked to sign the form on camera, if available, witnessed by the researcher remotely and by their care partner. Participants will be able to withdraw from the study at any point following consent, with no explanation required. Their participation or withdrawal from the study will not impact their regular care in any way.

#### 2.2.4 Sample size

The study will aim to recruit about 30 people living with dementia of different types and demographics in order to achieve a heterogeneous sample across different types of dementia, age and sex. There is no clear sample size guidance for cognitive interviewing. Previous think-aloud studies have used sample sizes in the region of 18–36 (Al-Janabi et al., 2013; Horwood, 2014; Bailey et al., 2016; Mitchell et al., 2020). The proposed sample size should be sufficient to reach “information power” in identifying important themes arising from interviews and enable the quantitative “scoring” element of the analysis (Malterud et al., 2016).

### 2.3 Instruments

#### 2.3.1 Cognitive assessment

*PROMIS-Cognitive Function- Short Form 4a (PROMIS-SF 4a)* (Physician Health Organization [PHO], 2020) is a 4-item instrument used to capture self-reported cognitive complaints. The PROMIS-SF 4a in this study will provide a subjective assessment of cognitive function by a person with dementia. This brief tool includes individual reviews of processing speed, working memory and executive function cognitive domains. Each question has five response options ranging in value from one to five. The sum of the response values to each question is added to generate a total raw score ranging from 4 to 20. Raw scores ranging between 15 and 20 are considered normal, mild complaints are between 12 and 14, moderate complaints are between 6 and 11, and severe complaints are less than 6 (P^®^ Scoring Manuals, 2022).

*General Practitioner Assessment of Cognition (GPCOG)* (Brodaty et al., 2002) is a screening tool for dementia, comprising two components: (1) cognitive test items conducted with the person and (2) historical questions asked of an informant. Results > 8 or < 5 on the GPCOG patient section indicate cognitively intact or impaired, respectively. For patients requiring an informant questionnaire, a score of 3 or less out of 6 in this section suggests cognitive impairment (Brodaty et al., 2002).

*The Dementia Communication Difficulties Scale (DCDS)* (Murphy et al., 2007) in this study will help further define a dementia stage, such as early, moderate, or late. DCDS comprises 13 statements based on existing definitions of the communication problems commonly experienced by people as dementia progresses. The DCDS requires a third party who knows the person with dementia well to assess various aspects of their communication on a 5-option scale. Each DCDS option is assigned a score: “Never” = 0, “Sometimes” = 1, “Often” = 2, “Always” or “Says too little for me to judge” = 3. A person’s DCDS rating is obtained by totaling their scores for all 13 statements. DCDS ratings can range from 0 to 39, with a higher rating indicating greater communication difficulty. Stages of dementia group definitions include: early stage (DCDS ratings between 0 and 10.5); moderate stage (DCDS ratings between 11 and 19.5); late stage (DCDS rating between 20 and 39) (Murphy et al., 2007).

#### 2.3.2 Quality of life

*The Quality of Life in Alzheimer’s Disease (QOL-AD)/AD-5D* (Logsdon et al., 2002) is a condition-specific instrument for assessing health-related quality of life for people living with dementia and the Alzheimer’s Disease Five Dimensions (AD-5D) (Comans et al., 2020) is a preference-based scoring algorithm for QOL-AD that calculates quality-adjusted life years and enables its use in economic evaluation. The QOL-AD/AD-5D comprises 13 attributes (physical health, energy, mood, living situation, memory, family, marriage, friends, self as a whole, ability to do chores, ability to do things for fun, money and life as a whole). Response options include 1 (poor), 2 (fair), 3 (good), and 4 (excellent), with higher scores indicating better quality of life. The patients’ ratings are performed in an interview, with standardized instructions to avoid influencing the results. It is worth noting that while the QOL-AD was originally developed as a disease-specific scale for Alzheimer’s, it has been used to assess the quality of life of people living with other types of dementia (Larsson et al., 2011).

*The Australian quality of life (AQOL-4D)* (Hawthorne et al., 1999) is a generic health-related quality of life instrument that consists of 12 attributes covering four dimensions, including independent living–self-care, household tasks and mobility; relationships–friendships, isolation and family role; mental health–sleeping, worrying and pain; and senses–seeing, hearing and communication. Each dimension has four response levels. It is an abbreviated version of AQOL questionnaire that could be completed in 1–2 min by a participant without an accompanying instructions (Hawthorne et al., 1999).

*The ICECAP-O* (Coast et al., 2008) is a capability wellbeing instrument developed for self-completion by older adults, specifically for the economic evaluation of health and care interventions. It consists of five dimensions relating to a person’s capability to have attachment, security, role, enjoyment, and control. Each dimension has four response levels ranging from no capability to full capability (Coast et al., 2008).

*The ICECAP-SCM* (Bailey et al., 2016) is a capability wellbeing instrument developed for use in evaluation of palliative and supportive care interventions. The instrument has seven attributes derived from qualitative data collected from those at various stages along the trajectory toward death. Participants are asked to indicate their wellbeing “at the moment” in terms of choice (being able to have a say), love and affection (being able to be with people who care about you), freedom from physical suffering, freedom from emotional suffering, dignity (being able to maintain dignity and self-respect), support (able to have help and support), and preparation (having the opportunity to make preparations). There are four response levels to each attribute ranging from no capability (1), a little capability (2), some capability (3), full capability, generally expressed as experiencing a lot of an attribute (4) (Huynh et al., 2017).

### 2.4 Data collection

The interview will begin with a recap of the study aims, interview format and data confidentiality, consent for participation and recording of the interview. In order to “warm up,” a preliminary task will ask participants to “think aloud” as they count windows at the place they live. Participants will also be asked a few basic questions about their age, dementia diagnosis, and their family circumstances. Participants will then be asked to reflect on what “good life” means to them and asked to verbalize their thoughts while completing the four PROMs, containing 37 questions in total, thinking aloud when reading and answering these questions. The order of the PROMs will be randomly determined prior to the interview, using a random number generated by RAND function in Microsoft Excel. Lastly, participants will be asked to self-rate their cognitive health with four standardized questions (Physician Health Organization [PHO], 2020); while a close person will be asked to complete DCDS and GPCOG.

Participants will not be interrupted unless they are silent for longer than 10 s when they will be asked to “keep thinking aloud.” A close person will be present and may offer support with interpretation during the interview if required, but they will be asked not to provide their own views. An interviewer will facilitate this interaction.

If the participant is able to provide consent but does not appear to be able to complete any of the PROMs, such as unable to directly participate, the interviewer will shorten the interview by thanking the participant for their interest in this study and sensitively terminating the interview. In such cases, we will not seek consent to interview a close person on behalf of a patient as the purpose of this study is specifically to examine the ability of people living with dementia directly to self-complete these PROMs and express their preferences.

After completing each PROM and after completing all four PROMs, there will be a short discussion to clarify the participants’ thoughts. The interviewer will seek to clarify any areas where there is uncertainty about the recorded answer and briefly discuss the participant’s opinions more generally.

Interviews could last anywhere between 1 and 1.5 h and will be audio recorded. The semi-structured part of the interview will be conducted using a topic guide to help ensure a consistent approach across interviews and between interviewers. However, the research team will use the guide in a responsive way tailored to individual experiences. This means that the topics covered and the order in which they are discussed could vary, especially between interviews. Interviewers will use open, non-leading questions, and answers will be fully probed (for example, asking “How?” and “In what way?”).

If participants become upset during the interview for any reason, the issue will be handled sensitively, and participants will be asked whether they would like to stop the interview. If the participant’s preference is to stop the interview, this will, of course, be done, but if they wish to continue, this preference will also be met. Following the facilitation of an interview, the researcher will be debriefed by another research team member. They will not share any information which can potentially identify the participant during the debrief.

### 2.5 Data analysis

All interviews will be transcribed verbatim. Key topics will be identified through familiarization with the transcripts and discussion among the research team to create a list of themes and sub-themes called “nodes.” The transcripts will be managed using the software package NVivo 10.

A central chart will be created to give an overview of each interview in terms of the key sampling characteristics. The final analytical stage will involve working through the coded data both within and across cases and themes, identifying similarities and differences and interrogating the data to seek to explain emergent patterns and findings (Spencer et al., 2013).

To explore differences in the completion of the PROMs, three raters will independently assess each transcript for errors in terms of:

• comprehension (understanding the question in the way intended);
• retrieval (retrieving information – in general, this is assumed to be the ability to retrieve information from long-term memory, but, for this case, with proxy respondents, it will also be used to indicate errors where the respondent is unable to retrieve information that they were unaware of);
• judgment (judging how the retrieved information should be used to answer the question);
• response (participant’s response is inconsistent with the personal experience expressed or the desired response is missing from the response choices); and,
• completion time.

Kappa scores will be calculated to assess inter-rater agreement, applying the following set of rules, used in the previous research by Froggatt et al. (2020), to determine whether a response should be classified as an error and, if so, of what type:

• If no error is identified by any rater, then no error will be recorded.
• If an error type is identified by all raters, then an error of that type will be recorded.
• If an error is identified by one or two raters, then the raters collectively will come to a final decision through discussion.
• If an error is identified by all three raters, but there will be disagreement about the nature of the error, then the raters will collectively come to a final decision through discussion.
• If raters cannot come to a collective decision through discussion, then the final assessment will be made based on majority choice.

### 2.6 Ethics

This study received approval by the St James’s Hospital and Tallaght University Hospital Joint Research Ethics Committee. Approval was granted on 11 April 2022.

### 2.7 Patient and public involvement (PPI)

In preparation for this study, we conducted extensive patient and public consultations. First, we engaged with the Dementia Community Research Advisory Panel members (DC-RAP) (Global Brain Health Institute [GBHI], 2022) established as part of the Global Brain Health Institute (GBHI) Person and Public Voice (PPV) project. We ran five one-on-one cognitive interviews with care partners of people living with dementia. Cognitive interviewing is an evidence-based, qualitative method specifically designed to investigate whether a questionnaire fulfill their intended purpose; often used as part of pretesting and before the main data collection (Willis, 2005).

During the cognitive interviewing, we obtained feedback on the topic guide, PROMs [ICECAP-O original and ICECAP-O “smiley version” (Kinghorn et al., 2021), AQOL-4D, DEMQOL-U, and QOL-AD], including their design, wording, and administration mode. Table 1 provides a synopsis of the questions asked, feedback received, and corresponding changes made to the instruments. Subsequently, an adapted version, underpinned by the AAC principles and accompanied by interviewer instructions was developed for the ICECAP-SCM and ICECAP-O.

**TABLE 1:** Synopsis of the PPI/PPV feedback received and corresponding changes made.

	ICECAP-O original	ICECAP-O “smiley version”	AQOL-4D	DEMQOL-U	AD-5D (QOL-AD)
Like most	Symmetrical, clean, consistent, easy to follow [PPI1–4]	Easy, simple, not busy, 1 page, quick [PPI1–4]	“Comprehensive” [PPI1]; “liked examples” [PPI1]; talked about “personal day to day tasks – easy to answer” [PPI1 and 4]; “gets down to emotional level… loneliness for example” [PPI2]	“colours delineate text” [PPI1]; “relatively simple” [PPI2]; “talked about feelings” [PPI4]	“covers QOL succinctly and add additional categories as marriage and money” [PPI2]; “not long” [PPI3]; “very clear” [PPI4]
Liked least	A lot of words and lack of space [PPI1–4]; “hard to answer” [PPI3 and 4] “emotionally draining” [PPI4]	“Colour – visually too much” [PPI2]; “too simple” [PPI1]	“a lot of words” [PPI1 and 4] and “acronyms” [PPI2]; “long” [PPI4]	“negatively worded questions (lonely, frustration)” [PPI2]; “kept forgetting about last week” reference [PPI1]; confusing words, i.e., cognition and forgetfulness, relationship defined as verbal communication [PPI3]; many questions are covered by other PROMs [PPI2]	“too many instructions” [PPI2 and 3]
Other comments	“Why can? It takes a minute to register; talking about yourself or your ability? If someone dependent – would they know that – depends on how advanced they are. Not all will be able to admit they lost independence” [PPI4]	Color and smile – double message (red-bad; green-good) what if a person prefers being dependent, but the associated color was red? [PPI4]	“If facilitated this would invite dialog, if given right away might scare them” [PPI1]; “Unlikely will answer over the past week, they will answer at the moment” [PPI4]		Questions about choirs and marriage may not apply to everyone (e.g., old fashioned gentleman, nursing home residents, widowed)
Changes made	Based on feedback, the lead author developed two alternative design options, i.e., visual, using bars and tabular; applied to both ICECAP-O and ICECAP-SCM, with the latter accompanied by interviewer instructions. A decision was made to use a tabular design in this study	A decision was made not to use this design in the present study	A decision was made to change some of the wording of questions, font size and spacing; removed abbreviations and acronyms. Approval was sought and granted to use this version in the present study	A decision was made not to use this PROM	No changes were made; a decision was made to use the original interviewer-led version in the present study

After the initial round of changes, we conducted another cognitive interview with a person living with dementia. We confirmed the chosen instruments, their design, and the likelihood of completing 37 questions across the four PROMs in an interview. Patient and public consultations during the analysis stage and dissemination will continue for the duration of this project.

## Ethics statement

The studies involving human participants were reviewed and approved by the St. James’s Hospital and Tallaght University Hospital Joint Research Ethics Committee. The patients/participants provided their written informed consent to participate in this study.

## Author contributions

IK wrote the initial draft, edited, and organized the final version of the manuscript. All authors provided expertise from their respective fields, involved in writing, review, and editing, and read and approved the final manuscript.
